# Multi-tissue transcriptomics for construction of a comprehensive gene resource for the terrestrial snail *Theba pisana*

**DOI:** 10.1038/srep20685

**Published:** 2016-02-08

**Authors:** M. Zhao, T. Wang, K. J. Adamson, K. B. Storey, S. F. Cummins

**Affiliations:** 1 School of Engineering, Faculty of Science, Health, Education and Engineering, University of the Sunshine Coast, Maroochydore DC, Queensland, 4558, Australia; 2 Institute of Biochemistry & Department of Biology, Carleton University, 1125 Colonel By Drive, Ottawa, ON, K1S 5B6, Canada

## Abstract

The land snail *Theba pisana* is native to the Mediterranean region but has become one of the most abundant invasive species worldwide. Here, we present three transcriptomes of this agriculture pest derived from three tissues: the central nervous system, hepatopancreas (digestive gland), and foot muscle. Sequencing of the three tissues produced 339,479,092 high quality reads and a global *de novo* assembly generated a total of 250,848 unique transcripts (unigenes). BLAST analysis mapped 52,590 unigenes to NCBI non-redundant protein databases and further functional analysis annotated 21,849 unigenes with gene ontology. We report that *T. pisana* transcripts have representatives in all functional classes and a comparison of differentially expressed transcripts amongst all three tissues demonstrates enormous differences in their potential metabolic activities. The genes differentially expressed include those with sequence similarity to those genes associated with multiple bacterial diseases and neurological diseases. To provide a valuable resource that will assist functional genomics study, we have implemented a user-friendly web interface, ThebaDB (http://thebadb.bioinfo-minzhao.org/). This online database allows for complex text queries, sequence searches, and data browsing by enriched functional terms and KEGG mapping.

With an estimated 75,000 living species, gastropod snails are among the most successful and diverse animal groups, found in terrestrial and aquatic ecosystems^1^. They are a critical component of our natural biodiversity; however, some species have invaded new areas where they give rise to significant environmental and economic problems. These snails damage native invertebrate fauna, act as intermediate parasite hosts, and cause multi-billion dollar financial losses as agricultural pests^2^. Despite extensive interest and funding directed towards traditional methods of invasive snail management (eg. toxic molluscicides), there is still no proven and efficient method of eliminating snails without detrimental effects to the surrounding ecosystem. For example, the white garden snail, *Theba pisana* (Helicidae family) originated from the Mediterranean and has now become a well-known agricultural and garden pests. It is known to cause destructive damage of numerous plants as well as economically important crops^3^. Nevertheless, the large-scale genomic data that is required for gene discovery is still not available for this and other land snail invasive species.

With the advance of DNA sequencing technologies, the generation of a large amount of sequence data from a non-model organism has become increasingly affordable^4^,^5^. Recently, we have provided a comprehensive neuropeptidome for *T. pisana* based largely on the transcriptome derived from three tissues: the central nervous system (CNS), hepatopancreas, and foot muscle^6^. To provide a comprehensive picture of their molecular biological processes, we present the first transcriptome-based database from three tissues of *T. pisana*. In total, clean reads were assembled using a two-step *de novo* assembly strategy, to provide a total of 250,848 unique transcripts for this well-known agriculture pest. Our further investigation of the protein-protein interaction network has revealed a highly connected interactome for *T. pisana*. Our transcriptome-based gene list maps the first genetic landscape for *T. pisana*, which advances our understanding on this invasive species towards targeted functional analyses. To share this valuable resource with the community to support further elucidation of molecular function, we present all the sequence and annotation data online at http://thebadb.bioinfo-minzhao.org/. This user-friendly web portal may assist researchers to design potential pest control strategies at the systems biology level, such as gene-gene interaction.

## Results

### Functional characterization of *Theba pisana* transcriptomes

**Figure 1:**
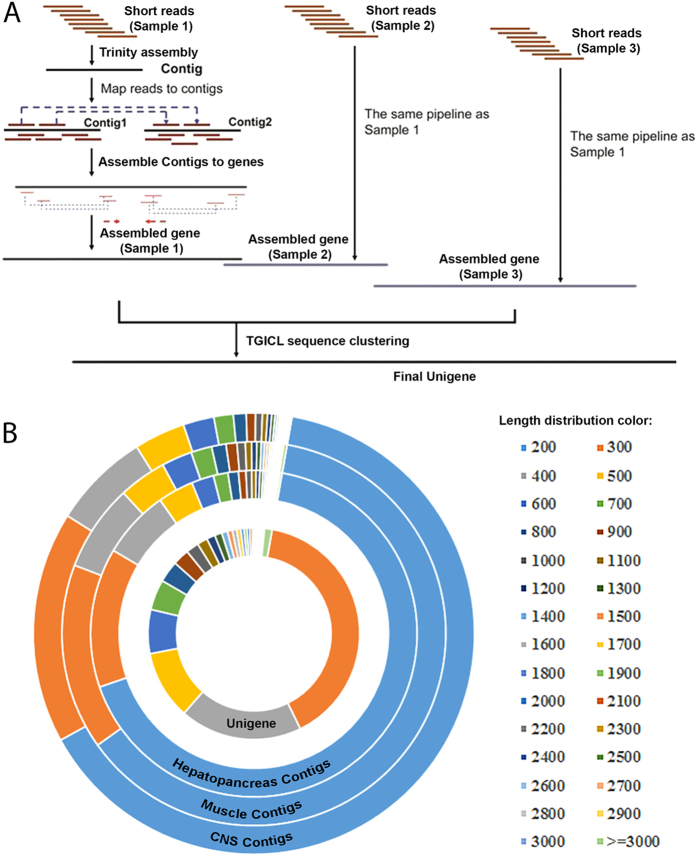
Workflow for transcriptome assembly and the length distribution for assembled contigs and unigenes. (**A**) The pipeline for transcriptome assembly. All the samples in this study were assembled separately and clustered to form unigenes. (**B**) The length distribution for contigs and unigenes. From inside out: the length of final unigene, the length of hepatopancreas transcriptome contigs, the length of foot muscle transcriptome contigs, the length of central nervous system (CNS) transcriptome Contigs.

Three normalized cDNA libraries derived from RNA isolated from CNS, hepatopancreas, and foot muscle were constructed and sequenced using the pair-end Illumina platform. These transcriptomes were assembled separately and clustered to form a unigene set (see Methods). Collectively, more than 339,479,092 raw reads with a total of 28,930,442,580 clean nucleotides were generated. The Q20 percentage is 98.45%, which represents the percentage of sequences with a sequencing error rate less than 1%. The average GC content for the three samples was 44.31%. The assembled transcriptomes represent a large number redundant, typically partial or isoform transcript sequences in different tissues. To produce larger and more complete consensus sequences, we utilized the TIGR Gene Indices clustering tools (TGICL)^7^ to further group those sharing transcripts of close identity (Fig. 1). As shown in Table 1, the three transcriptomes were assembled into 413,539 (CNS), 392,199 (hepatopancreas) and 377,830 (foot muscle) contigs. To characterize the assembly efficiency, we calculated the N50 length for each tissue as the length for which contigs of that length, or longer, contains at least half of the sum of the lengths of all contigs. The N50 of these three datasets is 283 (CNS), 321 (hepatopancreas) and 362 (foot muscle). Based on the assembled contigs, we further merged the overlapped contigs with TGICL. In total, 250,848 unigenes could be clustered from the three assembled contig datasets. Although the N50 of assembled contigs in the three transcriptomes is less than 400 bp, the N50 of the final clustered unigenes from the three transcriptomes is 712 bp. This substantial improvement in the sequence length highlights the effectiveness of extending the cDNA through utilizing multiple transcriptome datasets with this gene clustering approach.

**Figure 2:**
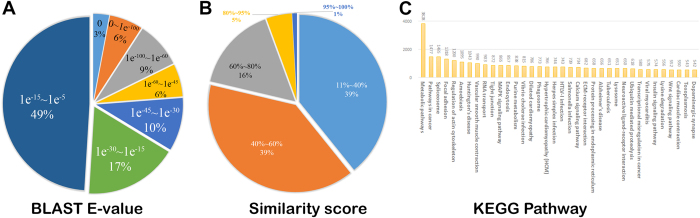
Functional annotation of unigenes in *Theba pisana*. (**A**) The BLAST E-value distribution; (**B**) the BLAST similarity score to known proteins; (**C**) KEGG pathway annotation.

To explore the associated biological processes of identified protein-encoding unigenes in *T. pisana*, various annotations were applied to the derived protein sequences. We first used a similarity search to map the unigene proteins to known proteins in the NCBI non-redundant protein database (Fig. 2A,B). In total, 52,590 proteins can be mapped to the Nr database. Almost half of the BLAST hits have a significant E-value (1e^−15^−1e^−5^), while about 39% of those hits have a 40 %–60 % similarity with known proteins. In addition, we can confidently map with high confidence 39,145 unigene proteins to the SwissProt database.

Using KEGG pathway analysis (Fig. 2C), we have annotated 33,208 unigenes in *T. pisana* using the BLAST E-value cutoff of 0.00001. This comprehensive pathway annotation not only helped to determine the number of relevant pathway genes but also provided their relative abundance ratio for different pathways. For example, there are 3,828 unigenes that annotate to specific KEGG metabolic pathways. For signaling pathways, the MAPK signaling pathway is most abundant with 865 annotated unigenes. Besides MAPK signaling, there are 734 unigenes within the calcium signaling pathway, 574 unigenes within the insulin-signaling pathway, and 552 unigenes associated with the Wnt signaling pathway.

To obtain a comprehensive insight into cellular function, gene ontology (GO) was performed, resulting in an annotation containing a total of 21,849 unigenes. For biological processes, molecular function and cellular component classes, there are 16,171, 17,515, 13,952 unigenes annotated, respectively. As shown in Figure S1A, 10,676 metabolic processes-related unigenes were detected, along with 6,611 unigenes classified within “biological regulation” and 6,116 unigenes within “response to stimulus”. Additionally, we found 12,173 unigene proteins with a binding function and 9,921 unigenes associated with catalytic activities (Figure S1B). Interestingly, there are 1,538 transporters and 767 receptors in the unigene set, which are predicted to function in metabolite, neuropeptide and other signaling biomolecule exchanges in *T. pisana*. These transporters may provide a basis for further systematic exploration into the snail transporter substrates^8^,^9^,^10^. Within the cellular component category, 8,749 unigenes sort into various intracellular organelles, while 5,348 unigenes associate with the membrane (Figure S1C).

For clusters of orthologous group (COG) database annotation, 15,575 protein-coding unigenes were mapped with a BLAST E-value cutoff of <1e^−5^ (Figure S1D). Approximately 6,140 of these can be categorized into the general cellular function class, while for other basic genetic processes, there are 2,433, 2,774, 2,299 unigene proteins that associate with transcription, translation, and replication, respectively. Other prominent categories include amino acid (1,213 unigenes), lipid (612 unigenes) and nucleotide metabolism (303 unigenes), which may be useful for the further investigation of metabolic regulation across multiple species^11^,^12^,^13^. There are few unigenes associated with extracellular structure (34 unigenes) and nuclear structure (12 unigenes) in our COG annotation.

With Pfam protein domain annotation, we searched for the presence of well-annotated protein domains based on the number of annotated unigenes (Table 2). In total, 1,126 unigenes annotate with a zinc finger domain, a well-known DNA-binding structure motif. Of those, 530 unigenes also annotate to zf-C2H2, which is a small motif known to accommodate one or more zinc ions and stabilize folding of a protein^14^. In addition, we found that other protein structure stabilization-related Pfam domains were abundant Pfam annotations, such as the ankyrin repeat (associated with 382 unigenes). The ankyrin repeat refers to a 33-residue motif in proteins containing two alpha helices separated by loops, which are critical for protein folding, stability and recognition^15^. Although the G protein–coupled receptor (GPCR) domains are not present in the top 20 annotated domains, there are 246 and 64 unigenes that annotate as 7-transmembrane receptors and GPCRs, respectively (Table S1). By combining these two, there are 258 unique unigenes that correspond to 7-transmembrane receptors or GPCRs.

### Identification of differentially expressed genes

**Figure 3:**
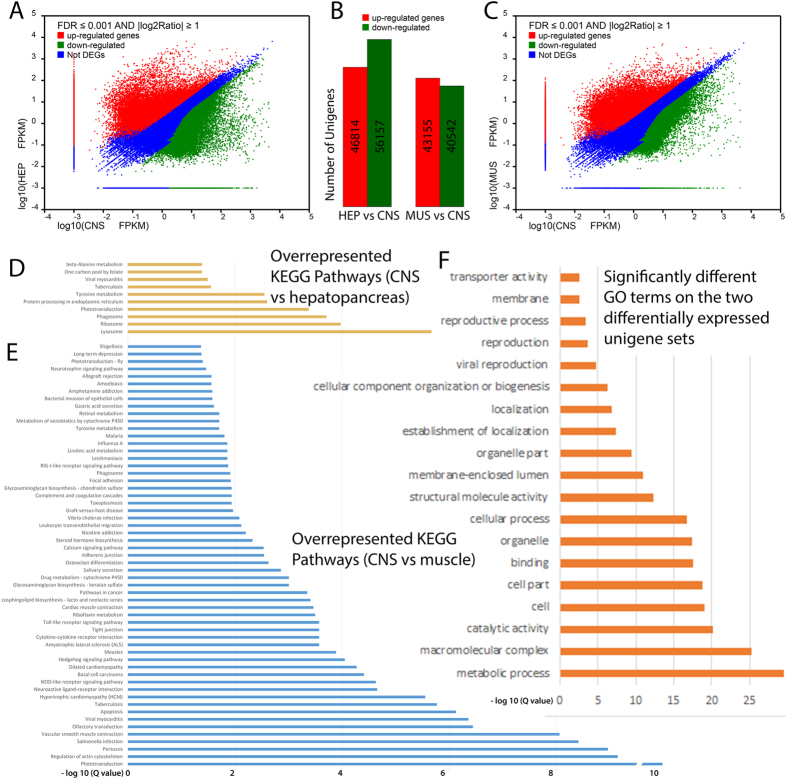
Differentially expressed genes in *T. pisana* CNS against hepatopancreas and foot muscle. (**A**) A density plot of differentially expressed genes in CNS to hepatopancreas, using the cutoff FDR less than 0.001 and the absolute value of log2 ratio greater than 1; (**B**) a density plot of differentially expressed genes in CNS to foot muscle, using the cutoff FDR less than 0.001 and the absolute value of log2 ratio greater than 1; (**C**) a comparison of up- and down-regulated genes between two gene sets. HEP, hepatopancreas and MUS, foot muscle; (**D**) The over-represented KEGG pathways in the differentially expressed genes from the CNS vs hepatopancreas; (**E**) The over-represented KEGG pathways in the differentially expressed genes from the CNS vs foot muscle; (**F**) The significantly different gene ontology terms in the two differentially expressed gene sets.

We explored *T. pisana* CNS-specific gene expression by comparing the CNS transcriptome separately against the hepatopancreas and foot muscle transcriptomes. Comparison of the CNS with hepatopancreas showed that 46,814 unigenes were more highly expressed in the CNS, while 56,157 unigenes showed relatively lower expression (Fig. 3A,B). In contrast, there are 43,155 highly expressed and 40,542 down-regulated unigenes in the CNS when compared to the foot muscle (Fig. 3B,C).

A gene-set enrichment analysis was adopted to characterize whether those CNS unigenes identified as differentially expressed had any significant annotations when compared to all unigenes in *T. pisana*. Using a cutoff at a corrected p-value less than 0.05, we identified 10 significantly enriched KEGG pathways from the 102,971 differentially expressed CNS unigenes when compared to the hepatopancreas (Table S2, Fig. 3D). However, the same threshold could obtain only 57 significantly enriched KEGG pathways for the 83,697 differentially expressed CNS unigenes when compared to the foot muscle (Table S3, Fig. 3E). There are four enriched pathways shared for the KEGG differentially expressed genes (phototransduction, tuberculosis, tyrosine metabolism and phagosome) which emphasise the unique biological functions of the CNS.

The number and majority of significantly enriched pathways is different in the other two transcriptome gene sets. The enriched pathways identified in the CNS compared to the hepatopancreas consist primarily of those related to metabolism, such as “one carbon pool by folate” and “beta-alanine metabolism”, which are a function of the snail hepatopancreas, a digestive gland that enables absorption of digested food. The enriched pathways present within the differentially expressed genes of the CNS against foot muscle are broadly relevant to various biological processes, which is reflective of large differences observed between the transcriptomes. It is worth noting that a few pathways are highly associated with CNS function, such as olfactory transduction. Also, multiple genes related to bacterial infection immunological response are enriched in the differentially expressed genes of the CNS against foot muscle, such as pertussis, shigellosis, and tuberculosis^16^,^17^,^18^. Pertussis is also commonly known as whooping cough, a highly contagious bacterial illness caused by *Bordetella pertussis*^16^, while shigellosis is a foodborne disease caused by infection by *Shigella* bacteria^17^. As a worldwide infectious fatal disease, tuberculosis is caused by various strains of mycobacteria, including *Mycobacterium tuberculosis*^18^. These enriched bacterial disease-related pathways in *T. pisana* tissues may provide clues for the potential interactions of bacteria with the snail.

Further comparative investigation of the two differentially expressed gene datasets (the 102,971 unigenes of CNS with foot muscle, and the 83,697 unigenes of CNS with hepatopancreas) confirmed their distinct functional distribution (Fig. 3F). Using the two combined unigene datasets as background, we utilized a Fisher’s exact test to evaluate the difference in shared GO terms, revealing a significant difference in metabolic processes (P value = 1.142498e^−206^). Among the 83,697 unigenes of the CNS compared to hepatopancreas, 5,098 annotate with metabolic processes. By comparison, 3,230 metabolic genes annotate from the 102,971 unigenes when comparing the CNS with foot muscle datasets. These results confirm that the three tissues have distinct metabolic and cellular regulatory activities.

### Construction of the first interactome in *T. pisana*

**Figure 4:**
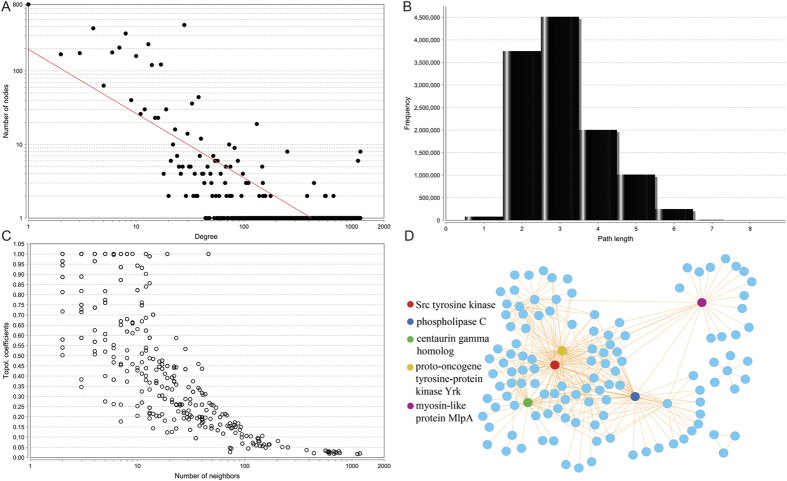
Constructed interactome of *Theba pisana*. (**A**) The degree distribution of the reconstructed interactome; (**B**) The short path distribution of the nodes in the network; (**C**) The topological coefficients of the reconstructed network; (**D**) The extracted synapse sub-network. The hub nodes are highlighted with different colors.

We annotated all the *T. pisana* unigenes with Pfam database and then utilized reliable public data resources for protein domain-domain interaction to construct a comprehensive interaction map for all the predicted proteins from the combined three tissue transcriptomes. The interactome constructed contains 3,913 genes that encode for proteins for which 41,653 protein-protein interactions are possible. Further network topological analysis indicates that most proteins in our map are not sparsely connected. On average, the number of neighbors for each node in the network is 21. As shown in Fig. 4A, there exists only 800 nodes with one connection, which means that the majority of nodes are highly inter-connected. The degree of all nodes in this protein-protein interaction network follows a power law distribution *P(k)* ~ *k*^−*b*^, where *P(k)* is the probability that a molecule has a connection with other *k* molecules, and *b* is an exponent with an estimated value of 0.872. This indicates that the reconstructed interactome of *T. pisana* differs from all the human PPI networks where most of the nodes are sparsely connected with exponent *b* as 2.9^19^. This feature made the shortest path distribution for the whole network skewed to 2 or 3, which means that the majority of protein connections can be reached in only 2 or 3 steps (Fig. 4B). This observation is confirmed by analyzing the relation between topological coefficients and the number of neighbors. As shown in Fig. 4C, the nodes with relatively low coefficients are more likely to have more neighbors. As there exists high modularity, the hub nodes in this network may have prominent roles as common connections to mediate rapid cellular signaling transduction pathways, which may in turn have biological benefits by enabling efficient responses to environmental signals.

Our functional predictions had collected 459 unigenes that annotate to neuron synapse structures in *T. pisana* (see Fig. 2F). Using our constructed interactome, we demonstrate the usefulness of a network-based data extraction approach to explore the synapse function (Fig. 4D). To extract a sub-network related to the synapse genes of interest, we used the Steiner minimal tree algorithm implemented in our previous studies^20^,^21^. In this algorithm, all input genes were mapped to the *T. pisana* interactome. Finally, a minimum sub-network of synapse genes connected by their shortest path was produced. In this network, we highlighted five hub nodes with a high number of connections. Two of the five hub nodes (Src tyrosine kinase and proto-oncogene tyrosine-protein kinase) centered in the network are closely related to synapse function. The Src tyrosine kinase enzymes have a role in phosphorylating synapsin to regulate neurotransmitter release^22^. The tyrosine kinase is a critical protein involved in synapse remodeling, related to learning and memory^23^. In summary, our interactome constructed from *T. pisana* unigenes provides a broad, highly modular structure of cellular signaling pathways in *T. pisana* and can now be used for further exploration of specific biological processes.

### A web interface for *T. pisana*

**Figure 5:**
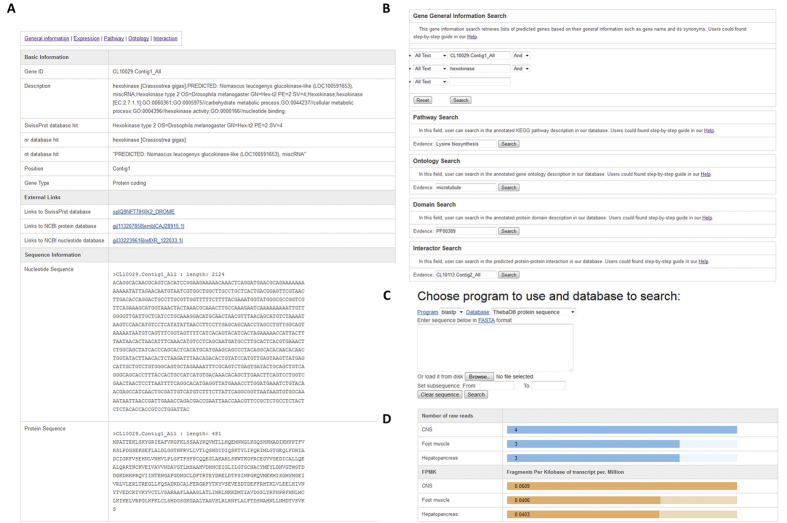
Web interface of ThebaDB. (**A**) An example of basic information shown in each unigene page; (**B**) Query interface for text search; (**C**) BLAST search interface for comparing query against all sequences in ThebaDB; (**D**) An example gene expression profile for a unigene with number of raw reads and calculated FPKM (Fragments Per Kilobase of transcript per Million mapped reads).

The ThebaDB was set up to be freely accessible at http://thebadb.bioinfo-minzhao.org/. In ThebaDB, all Unigene IDs are used as key, which enables comprehensive hyperlinks to various annotations (Fig. 5A). For all the genes in ThebaDB, we provided five sub-pages to characterize five annotation categories, including the general gene sequence information, the tissue expression profile, the biological process, the protein domain, and predicted protein-protein interaction information. For example, we highlighted their relevant genes with a red color in the corresponding pathway map for the annotated KEGG pathway related to each gene.

To help users perform text queries against our ThebaDB data, we developed five powerful query forms associated with general information, gene ontology, KEGG pathway, protein domain, and protein interaction (Fig. 5B). Notably, a quick text search for Unigene ID, and gene information is found at the top right of each page, which is useful to quickly access any data in the database. In addition, we deployed a sequence similarity search (BLAST) web interface for all the nucleotide and protein sequences in ThebaDB (Fig. 5C). Moreover, users can browse the transcriptome data using a web browser; including the top annotation gene ontology terms, Pfam domains and the annotated KEGG pathways. By providing the marked KEGG pathway chart with the *Theba* genes, users obtain a global view for each annotated KEGG pathway. By incorporating the gene expression profile from three tissues in this study, we also generated a raw read mapping and FPKM (Fragments Per Kilobase of transcript per Million mapped reads) bar chart to present an expression overview for three tissues (Fig. 5D). Finally, for the purpose of advanced data usage, we provide downloadable sequences corresponding to all the unigenes. The basic annotation for all the unigenes is also provided as a plain text .

## Conclusion and Discussion

Invasive snails are a constant and intractable pest of native fauna and agriculture crops, disrupting fragile ecosystems as well as destroying beneficial plants. Central to their success are molecular mechanisms that control and coordinate essential biological processes such as immune defense, reproduction and hypometabolism (hibernation or aestivation), as a consequence of unknown molecular events. To fully comprehend the complexity and functioning of these snails, genomic resources are paramount. Our newly assembled transcriptome data yielded 250,484 unique genes with an average length of 557 bp, which provides a valuable genomic resource to assist studies on functional genomics of this invasive species. Based on the generated data, we built a user-friendly web interface database, ThebaDB, to enable users to rapidly search and retrieve annotated genes.

The total of 250,484 unigenes was achieved by adopting a two-step assembly approach to cluster the unigenes. We assembled the three transcriptomes separately and clustered all the contigs to form unigenes; the final unigenes have longer sequences in average. We further searched the open reading frames (ORFs) based on the clustered RNA sequences. After mapping to the known proteins in NCBI Nr database, we found that only 20.96% of the predicted ORFs had a corresponding homolog. The majority of the ORFs would require further evidence to support their coding potential. Our further BLASTCLUST analysis showed that many of the sequences could be grouped, which may represent protein families. Since we have a limited number of tissue transcriptomes, assembly of all three transcriptomes together may provide an alternative approach to remove potential redundancies.

Our previous study provided a global molecular insight into the neuropeptides of *T. pinasa*^6^. We now provide an in-depth bioinformatics analysis of the transcriptomes, from annotation, functional classes of proteins, differential expression of all genes, proposed interaction and the development of an online web interface for research to rapidly curate the land snails gene data. For example, using the sequence-based homolog mapping, we found that many human cancer-related homologous genes are present, including 1,477 unigenes that annotate to “pathways in cancer”. Moreover, numerous disease-related pathway homologous genes are also abundant in *T. pisana*. For example, there are 1,043 and 656 unigenes associated with Huntington’s disease and 656 with Alzheimer’s disease. These homologous disease-associated genes may be useful for the evolutionary study of cancer gene function^24^ and conserved gene regulatory interaction patterns^25^.

The vertebrate learning system is generally believed to depend on complex events involving both presynaptic and postsynaptic cellular changes^26^. It is recognized that the CNS of molluscs is distinct from that of vertebrate nervous systems^27^ and molecular neural studies performed on the aquatic mollusca *Aplysia californica*, have revealed that synaptic plasticity is mediated exclusively through presysnaptic mechanisms^28^. Although there is accumulating evidence for synaptic plasticity in these aquatic molluscs, the function and regulatory mechanisms of synapse machinery in land molluscs has not been explored systematically. In this study, the predicted interactome generated for *T. pinasa*, pinpoints two hub genes related to synapse function; the Src tyrosine kinase and proto-oncogene tyrosine-protein kinase. This suggests that a similar synaptic toolkit exists within the CNS of land snails. However, the spatial localization of these two genes within the region of neural presynapse or postsynapse deserves further experimental validation to confirm a similar synaptic-associated function to that of *A. californica*. We only explored the synapse network and further systems biology-based approaches would be useful to explore the metabolic network in the land snail^11^,^12^,^13^,^29^.

In this study, we presented a differentially expressed gene analysis that has identified global differences between tissue transcriptomes of *T. pinasa*. This constitutes a first comprehensive investigation into the genetic landscape for an invasive snail species that can support further investigations of molecular function.

## Methods

### Tissue collection and assembly of transcriptome data for *Theba pisana*

To obtain a comprehensive protein-coding dataset, snails (*Theba pisana*) were collected in in early spring (September) at agricultural sites on the Yorke Peninsula, South Australia^6^. Three normalized cDNA libraries derived from RNA isolated from CNS, hepatopancreas, and foot muscle were constructed and sequenced using the pair-end Illumina platform^6^. To harvest the comprehensive transcriptome, we combined RNAs from three different metabolic states for each tissue, including active, waking and aestivating snails^6^. All the coding RNAs were extracted from tissue using TRIzol Reagent (Invitrogen) following the manufacturer’s protocol. Those extracted RNAs were further purified using oligo-dT and fragmented for complimentary DNA (cDNA) synthesis. Furthermore, PCR amplification were used to construct the cDNA libraries using random hexamer primed cDNAs. All the samples were sequenced using an Illumina HiSeq 2000 sequencing platform (BGI, Hong Kong). In total, 22.3 Gb pair-end with 100bp raw reads were generated and submitted to the NCBI Sequence Read Archive (SRA) accession SRP056280 for public use. The short reads were further trimmed to remove the low quality sequences. For each tissue, the trimmed reads were used for *de novo* assembly using Trinity^30^. The assembled contigs were further assembled to genes in each tissue sample. Since we had three tissues, we adopted a bioinformatics TGICL^7^ to cluster all the assembled sequences from each tissue to form a non-redundant unigene dataset. In our database, the unigene IDs were divided into two classes, clusters with the prefix CL, and singletons with the prefix Unigene.

### Differentially expressed Unigenes and protein-coding prediction

For all the detected unigenes, we calculated their gene expression using the FPKM method, which represents the fragments per kilobase of transcript per million fragments mapped. The formula used to assign FPKM is:


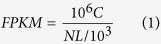


Assigns FPKM (*g*) to be the expression of gene *g*; C represents the number of fragments that uniquely aligned to gene *g*; N is the total number of fragments that uniquely aligned to all genes; and L indicates the number of bases on gene *g*.

To further detect the differentially expressed Unigenes, we used DESeq to analyze differentially expressed genes. Since we only have one biological sample for each tissue, we assumed that the mean expression score is a reliable predictor for the dispersion. Based on this assumption, DESeq estimated the dispersion from comparing the FPKM across conditions as ersatz for a proper estimate of the variance across multiple replicates. This approach was also assumed that most genes to behave the same within replicates as across conditions. Therefore, the differentially expressed genes will only cause the dispersion estimate to be too high. By applying the DESeq with a thresholds of FDR Q value  < 0.05 & log2 (fold change) >1, we defined the differentially expressed Unigenes.

To obtain the protein-coding sequence from the assembled Unigene nucleotide sequence, we performed protein-coding prediction using OrfPredictor^31^ by default parameters on the unigenes. For accuracy, we only retained the predicted longest ORFs and removed those amino acid sequences less than 30 amino acids. In total, 248,374 out of 250,848 unigenes were translated into a protein sequence.

### Biological functional annotations and database construction

To characterize the predicted biological function for all the identified unigene proteins, we annotated the proteins using protein sequence similarity, KEGG Pathway^32^, COG^33^, Gene Ontology (GO)^34^, and Pfam protein domain^35^. We searched All-Unigene sequences against three protein databases including the NCBI non-redundant database (Nr)^36^, SwissProt protein database^37^, KEGG pathway database^32^, and using BLASTX (cutoff E-value  < 0.00001). We predicted protein function by extracting annotations from the most similar protein in those databases. In detail, the KEGG pathway database contains well-annotated biological processes in the cells, and variants of them specific to particular organisms. KEGG pathway-based analysis can assist users to further understand the biological functions of genes on a pathway level. The COG database classified all orthologous gene products across system evolution relationships of bacteria, algae and eukaryotes^33^. All the proteins in COG are assumed to have evolved from an ancestral protein, and the whole database is built on protein coding sequences from those complete genomes. The unigenes in our database were aligned to the COG database to predict and classify possible functions. Using BLAST2GO^38^, we assigned GO functional annotation by BLAST against Nr protein databases. As an international standardized gene functional classification system, GO offers a dynamic-updated controlled vocabulary to comprehensively describe properties of genes and their products. To provide an overview for the biological function, we annotated all the predicted proteins using the Pfam database (version 27.0)^35^. Using all the HMM profiles related to 14,836 protein domains, HMMSEARCH^39^ was used to associate proteins with Pfam domains. We used the threshold of E-value as 0.00001 to identify reliable hits.

### Predicting a protein-protein interaction network using protein domain-domain interaction data

To present a protein-protein interactome for *T. pisana*, we predicted protein-protein interaction using the domain-domain interaction. To achieve this, we first adopted the HMMER^39^ to annotate all the known protein domains based on Pfam databases^35^. Based on the annotated protein domains, the domain-domain interactions from the DOMINE database (download on July 20^th^, 2014)^40^ were used to connect those proteins with domain-domain interaction relationship. The final network visualization and topological properties were generated by using Cytoscape (version 2.8)^41^.

The topological features of biological networks are useful for characterizing the potential function^42^. To explore the potential function of our reconstructed interactome, topological analyses were conducted using the NetworkAnalyzer plugin in Cytoscape (Fig. 3b–d)^41^. The degree was defined as the number of connections for each node in our network^42^. The betweenness centrality of network was calculated using the proportion of the nodes locating on shortest paths between two other nodes^42^. The topological coefficient in this study was used as a relative measure for the extent to which a node shares neighbors with other nodes^42^.

### Web interface development

To provide a web interface for the public to access our *T. pisana* transcriptomes, we managed all the data and annotations using the relational database management system MySQL. A user-friendly web interface was developed to read and browse the database using the Perl CGI module and JavaScript technology. The apache web server on a Linux server was used to publish the web pages dynamically.

## Additional Information

**How to cite this article**: Zhao, M. *et al.* Multi-tissue transcriptomics for construction of a comprehensive gene resource for the terrestrial snail *Theba pisana*. *Sci. Rep.* **6**, 20685; doi: 10.1038/srep20685 (2016).

## Supplementary Material

Supplementary Information

Supplementary Dataset 1

Supplementary Dataset 2

Supplementary Dataset 3

## Figures and Tables

**Table 1:** The assembly statistics for three tissues in ThebaDB.

	Sample	Total Number	Total Length (nt)	Mean Length (nt)	N50 (nt)
Contig	CNS	413,539	99,427,155	240	283
	Hepatopancreas	392,199	98,617,325	251	321
	Foot muscle	377,830	102,711,825	272	362
Unigene	CNS	–	86,214,554	391	457
	Hepatopancreas	–	85,422,670	459	605
	Foot muscle	–	96,749,312	480	655
	All	250,848	139,773,058	557	712

**Table 2:** The top 20 abundant Pfam domains in ThebaDB.

Pfam name	Pfam ID	Number of unigenes	Description (related gene ontology)
zf-H2C2_2	PF13465.1	542	Zinc-finger double domain
zf-C2H2	PF00096.21	530	Zinc finger, C2H2 type; metal ion binding (GO:0046872)
Pkinase	PF00069.20	514	Protein kinase domain; protein phosphorylation (GO:0006468)
Pkinase_Tyr	PF07714.12	453	Protein tyrosine kinase; protein phosphorylation (GO:0006468)
zf-C2H2_4	PF13894.1	449	C2H2-type zinc finger, type 4
Ank_2	PF12796.2	390	Ankyrin repeats (3 copies)
Ank	PF00023.25	382	Ankyrin repeat
Ank_4	PF13637.1	357	Ankyrin repeats (many copies)
Ank_5	PF13857.1	346	Ankyrin repeats (many copies)
Ank_3	PF13606.1	336	Ankyrin repeat
LRR_4	PF12799.2	325	Leucine Rich repeats (2 copies); protein binding (GO:0005515)
RRM_1	PF00076.17	322	RNA recognition motif. (RRM, RBD, or RNP domain)
LRR_8	PF13855.1	320	Leucine rich repeat; protein binding (GO:0005515)
WD40	PF00400.27	301	The beta-transducin repeat
RRM_6	PF14259.1	293	RNA recognition motif (RRM, RBD, or RNP domain)
7tm_1	PF00001.16	246	The 7 transmembrane receptor (rhodopsin family)
Ras	PF00071.17	241	Ras family; small GTPase mediated signal transduction (GO:0007264)
I-set	PF07679.11	237	Immunoglobulin I-set domain;
LRR_1	PF00560.28	231	Leucine Rich Repeat; protein binding (GO:0005515)
EF-hand_1	PF00036.27	229	EF hand, helix-loop-helix structural domain or motif found in a large family of calcium-binding proteins; calcium ion binding (GO:0005509)
