# Supplementary material for: Multi-tissue transcriptomics for construction of a comprehensive gene resource for the terrestrial snail Theba pisana
Source: Sci Rep. 2016 Feb 8;6:20685. doi: 10.1038/srep20685 (PMC4745086; doi:10.1038/srep20685)
Supplement: Supplementary Information [file srep20685-s1.pdf]

Supplement Figure

Multi-tissue transcriptomics for construction of a comprehensive gene resource for the terrestrial snail *Theba pisana*

M. Zhao[1], T. Wang[1], K.J. Adamson[1], K. B. Storey[2], S.F. Cummins[1,§]

School of Engineering, Faculty of Science, Health, Education and Engineering, University of the Sunshine Coast, Maroochydore DC, Queensland, Australia, 4558; [2] Institute of Biochemistry & Department of Biology, Carleton University, 1125 Colonel By Drive, Ottawa, ON, Canada K1S 5B6

[§] To whom correspondence should be addressed, Scott Cummins, scummins@usc.edu.au.

Figure S1. Functional annotation of unigenes in Theba pisana using the gene ontology three functional domains: (A) biological process, (B) Molecular function, and (C) Cellular component; and (D) NCBI Clusters of Orthologous Groups of proteins (COG).

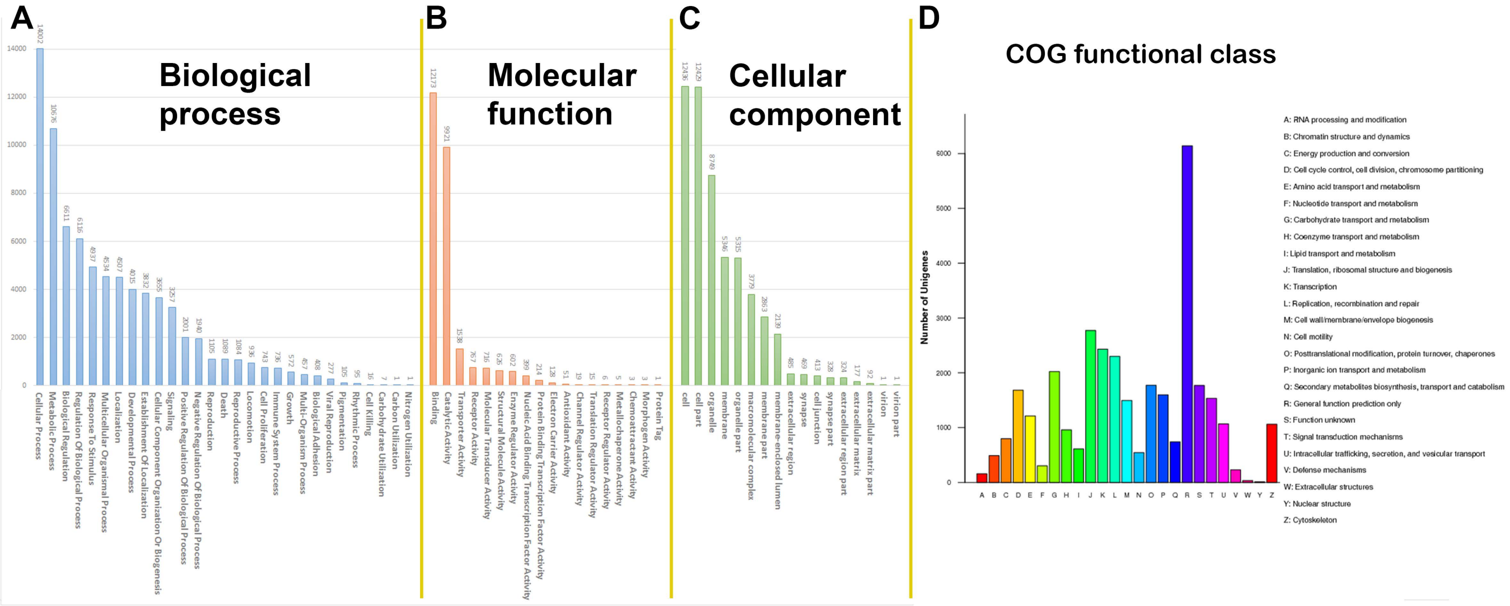

A
Biological process
14002
10676
6611
6116
4937
4534
4507
4015
3832
3655
3257
2001
1940
1105
1089
1084
936
743
736
572
457
408
277
105
95
16
7
1
1
Cellular Process
Metabolic Process
Biological Regulation
Regulation Of Biological Process
Response To Stimulus
Multicellular Organismal Process
Localization
Developmental Process
Establishment Of Localization
Cellular Component Organization Or Biogenesis
Signaling
Positive Regulation Of Biological Process
Negative Regulation Of Biological Process
Reproduction
Death
Reproductive Process
Locomotion
Cell Proliferation
Immune System Process
Growth
Multi-Organism Process
Biological Adhesion
Viral Reproduction
Pigmentation
Rhythmic Process
Cell Killing
Carbohydrate Utilization
Carbon Utilization
Nitrogen Utilization
B
Molecular function
12173
9921
1538
767
716
626
602
399
214
128
51
19
15
6
5
3
3
1
Binding
Catalytic Activity
Transporter Activity
Receptor Activity
Molecular Transducer Activity
Structural Molecule Activity
Enzyme Regulator Activity
Nucleic Acid Binding Transcription Factor Activity
Protein Binding Transcription Factor Activity
Electron Carrier Activity
Antioxidant Activity
Channel Regulator Activity
Translation Regulator Activity
Receptor Regulator Activity
Metallochaperone Activity
Chemoattractant Activity
Morphogen Activity
Protein Tag
C
Cellular component
12436
12429
8749
5346
5315
3779
2863
2139
485
469
413
328
324
177
92
1
1
cell
cell part
organelle
membrane
organelle part
macromolecular complex
membrane part
membrane-enclosed lumen
extracellular region
synapse
cell junction
synapse part
extracellular region part
extracellular matrix
extracellular matrix part
virion
virion part
D
COG functional class
Number of Unigenes
A: RNA processing and modification
B: Chromatin structure and dynamics
C: Energy production and conversion
D: Cell cycle control, cell division, chromosome partitioning
E: Amino acid transport and metabolism
F: Nucleotide transport and metabolism
G: Carbohydrate transport and metabolism
H: Coenzyme transport and metabolism
I: Lipid transport and metabolism
J: Translation, ribosomal structure and biogenesis
K: Transcription
L: Replication, recombination and repair
M: Cell wall/membrane/envelope biogenesis
N: Cell motility
O: Posttranslational modification, protein turnover, chaperones
P: Inorganic ion transport and metabolism
Q: Secondary metabolites biosynthesis, transport and catabolism
R: General function prediction only
S: Function unknown
T: Signal transduction mechanisms
U: Intracellular trafficking, secretion, and vesicular transport
V: Defense mechanisms
W: Extracellular structures
Y: Nuclear structure
Z: Cytoskeleton
